# Water buffalo milk: physicochemical, nutritional properties, and potential benefits for human health

**DOI:** 10.3389/fnut.2026.1742552

**Published:** 2026-04-24

**Authors:** Daniel Mota-Rojas, Fabio Napolitano, Arthur Fernandes Bettencourt, Andrea Bragaglio, Eleonora Nannoni, Lydia Lanzoni, Alfonso Chay-Canul, Adolfo Álvarez-Macías, Adriana Domínguez-Oliva, Vivian Fischer, Ayman H. Abd El-Aziz, Julio Martínez-Burnes, Ismael Hernández-Avalos, Patricia Mora-Medina, Adriana Olmos-Hernández, Fabiola Torres-Bernal, Nancy Jose, Ada Braghieri

**Affiliations:** 1 Neurophysiology, Behavior and Animal Welfare Assessment, DPAA, Universidad Autónoma Metropolitana (UAM), Mexico City, Mexico; 2 Scuola di Scienze Agrarie, Forestali, Alimentari ed Ambientali, Università degli Studi della Basilicata, Potenza, Italy; 3 Department of Animal Science, Federal University of Santa Maria, Santa Maria, Rio Grande do Sul, Brazil; 4 CREA Research Centre for Engineering and Agro-Food Processing, Consiglio per la Ricerca in Agricoltura el’Analisi dell’Economia Agraria, Treviglio, Italy; 5 Exo Research Organization, Potenza, Italy; 6 Department of Veterinary Medical Sciences, DIMEVET, University of Bologna, Bologna, Italy; 7 Animal Production and Health Division, Food and Agriculture Organization (FAO), Rome, Italy; 8 División Académica de Ciencias Agropecuarias, Universidad Juárez Autónoma de Tabasco, Villahermosa, Mexico; 9 Department of Animal Science, Federal University of Rio Grande do Sul, Porto Alegre, Rio Grande do Sul, Brazil; 10 Department of Animal Husbandry and Animal Wealth Development, Faculty of Veterinary Medicine, Damanhour University, Damanhour, Egypt; 11 Instituto de Ecología Aplicada, Facultad de Medicina Veterinaria y Zootecnia, Universidad Autónoma de Tamaulipas, Ciudad Victoria, Mexico; 12 Facultad de Estudios Superiores Cuautitlán, FESC, Universidad Nacional Autónoma de México (UNAM), Cuautitlán, Mexico; 13 Division of Biotechnology, Instituto Nacional de Rehabilitación Luis Guillermo Ibarra Ibarra (INR-LGII), Mexico City, Mexico

**Keywords:** ACE-inhibitory peptides, anticancer, bioactive compounds, casein micelle structure, milk fat globule membrane

## Abstract

Water buffalo (*Bubalus bubalis*) milk is the second most important dairy source worldwide. It is characterized by a higher content of total solids, fat, protein, calcium, and phosphorus than *Bos taurus* and *Bos indicus* milk. Its physicochemical properties include high viscosity, buffer capacity, thermal stability, and a lower freezing point, attributes that favor its conservation and industrial processing. Likewise, its lipid profile, rich in triacylglycerides and fatty acids such as palmitic, oleic and stearic, contributes to improving the texture and quality of dairy products, while its exclusive A2 *β*-casein content, together with high levels of antioxidant vitamins (A, C, E and B12), minerals (Ca, P, Mg, Zn) and bioactive peptides with antioxidant, anti-inflammatory, antihypertensive and immunomodulatory effects, reinforces its potential as a functional food. This review aims to integrate this evidence to provide a unified overview that serves as a basis for future research, technological development, and the optimization of buffalo milk use in nutrition and the dairy industry. Buffalo milk has shown significant anti-inflammatory activity due to the presence of peptides and the reduction of reactive oxygen species. Natural antioxidants present in buffalo milk have been shown to neutralize free radicals and significantly inhibit oxidative activity. Moreover, the presence of protein hydrolysates and *α*-glucosidase inhibitors can reduce blood serum glucose levels, as well as cholesterols and triacylglycerol levels, suggesting its anti-diabetic properties. Finally, buffalo milk has potential implications on bone metabolism, gastrointestinal health, and antineoplastic processes related to its high content of calcium, presence of lactic acid bacteria, and participation in cytotoxicity and reduced cell viability, respectively.

## 1 Introduction

Water buffalo (*Bubalus bubalis*) milk is the second-largest dairy product worldwide, accounting for approximately 15% of the total milk produced in 2022 (1). This represents approximately 150.341 million tons of milk annually. The leading producing nations include India (65%), Pakistan (30%), China (4%), Egypt (<1%), and Nepal (<1%) (2, 3). The vast majority of the global buffalo population (approximately 98%) is concentrated in South Asia, where 96.6% of the world’s buffalo milk is produced (4, 5). However, in South America, the Amazon Basin in northern Brazil houses at least 1 million water buffalo heads, representing 14.06% (75 million liters) of the country’s annual buffalo dairy production. Although the majority of buffalo are currently used for meat production in India, the dairy industry is actively developing (5, 6). The predominant dairy breeds of water buffalo are Murrah, Nili-Ravi, Surti, Mediterranean, and Kundi, breeds with daily milk yields around 8.27–9.77 kg/day/head. As a comparison, dairy cattle adapted to tropical climates yield on average 20.24–25.67 kg/day/head (4).

Beyond Asia, recent field data from southern Brazil underscore compositional heterogeneity and value-adding opportunities in buffalo milk; herd-level fatty-acid profiles show considerable proportions of desirable fatty acids and responsiveness to pasture and supplementation strategies, with direct technological and nutritional implications for local dairy chains (7).

Several processed products can be made from buffalo milk, including cheese, yogurt, butter, and other fermented products (8, 9). The global production of buffalo milk products, such as fresh and processed cheeses, has increased exponentially since 2010, reaching 630,000 tons by 2023. The leading producers are Africa and Europe with 51 and 44%, respectively (2). Italy, with the protected designation of origin “mozzarella di bufala campana,” represents an industry valued at hundreds of millions of euros annually (10). On the other hand, products such as ghee and butter are also derivatives of buffalo milk. They are widely consumed in the Asian continent with annual production of 4 million tons of ghee and 1 million tons of butter (1, 2, 11), of which 96.6% is produced in South Asia. In the Asian-Pacific region in particular, annual buffalo milk production exceeds 55 million tons, with India and Pakistan being the largest producers worldwide. In 2019, India recorded its highest production of buffalo milk, reaching approximately 92 million tons (12).

Most research regarding the physicochemical and nutritional properties of milk has been performed in cattle. In recent years, research on water buffalo milk has advanced significantly in characterizing its lipid and protein profiles. However, research has shown that from a biological perspective, buffalo milk is notable for its high concentration of total solids, fats, proteins, calcium, and phosphorus, making it especially suitable for the production of high-value dairy products such as mozzarella, ghee, paneer, and other traditional dairy products (1, 13, 14). When compared to the milk of other species such as camel, buffalo milk has higher fat, protein, lactose, and total solid content (15, 16). Water buffalo milk has a greater technological and nutritional value due to its physicochemical parameters (17). Among them, the viscosity values of Murrah buffalo milk (1.5 and 1.8 centipoise) stand out due to its higher content of total solids (mainly fat and proteins) (18), differing from *Bos taurus* and *Bos indicus* milk (1.53–1.65 centipoise) (1). Likewise, the freezing point of Murrah buffalo milk has average values of −0.552 to −0.528 °C due to its higher solute concentration, allowing it to be more stable to cold storage processes (1, 19). Moreover, its buffer capacity is also considerably high (0.042), due to the abundant protein and mineral concentrations, allowing it to tolerate pH variations during fermentation processes (18, 19). Buffalo milk also has higher content of lipids, proteins (20), bioactive peptides, vitamins, and antioxidants. Organoleptic characteristics such as a denser and creamier texture implies the increased quality of high-performance dairy derivatives such as cheese, ghee, and yogurt (17, 21).

Despite these advances, research on water buffalo milk has significant limitations. First, most available studies have focused almost exclusively on compositional, technological, or nutritional aspects, without an integrated approach that would allow for a comprehensive understanding of its potential as a raw material. Additionally, there is limited standardized comparison with cattle milk (*Bos taurus* and *Bos indicus*), making it difficult to objectively assess differences and advantages. Therefore, this review adopts an integrative approach that addresses the relationship between the molecular structure of its components (proteins, lipids, and bioactive components) and their impact on the quality of dairy products and human health. Furthermore, a comparison with cattle milk is included using the same parameters, allowing for the identification of real competitive advantages in terms of industrial yield, physicochemical stability, and nutritional value. This comparative approach contributes to a better appreciation of buffalo milk as an enhanced raw material.

Currently there is an increasing interest in adopting alternative food options to improve human health or functional foods, defined as “A food, which beneficially affects one or more target functions in the body, beyond adequate nutritional effects, in a way that is relevant to either an improved state of health and wellbeing and/or reduction of risk of disease” (22, 23). An example is the antioxidant and anti-inflammatory properties of water buffalo milk. Due to its nutritional and bioactive profile, buffalo milk is considered more nutritious than dairy cattle milk (19, 24). However, the integration of the functional food concept with the physicochemical and bioactive characterization of buffalo milk is still in early stages, representing a key research opportunity. This review positions buffalo milk within the framework of functional foods, establishing links between its bioactive components (e.g., vitamins, bioactive peptides, among others) and their potential health effects, thus identifying opportunities for the development of innovative, value-added products in the dairy industry. This review aims, by integrating this statement, to provide a unified overview that serves as a basis for future research, technological development, and the optimization of buffalo milk use in nutrition and the dairy industry. In the text, the term “conventional cattle” will be used when referring to *Bos taurus* and *Bos indicus* information.

## 2 Physicochemical characteristics of buffalo milk

Water buffalo milk is rich in solid components, nutrients, and functional properties that differentiate it from conventional cattle milk. In terms of composition, it has a high total solid content of 16 to 18%, exceeding the average for conventional cattle milk (<15%). This characteristic increases the energy value and enables superior yield in the production of dairy products (17, 25, 26).

Regarding its fundamental physicochemical characteristics, its density and specific gravity range from 1.030–1.037 g/mL indicate a higher concentration or molecular weight of dissolved solids (fat and solid-not-fat). In particular, Ahmad et al. (25) estimated an average density of 1.034 g/mL in Murrah breed buffalo milk and 10.2% solid-not-fat (SNF). However, according to the reports of Godinho et al. (6), in Brazilian herds, variation in density is associated with the season. During the summer, density increased up to 1.0351 g/mL, while a reduction was observed in autumn. Moreover, these values are positively correlated with the solid concentration. This is similar to what Sales et al. (27) found in water buffalo in Northern Brazil, where density was 1.033 g/mL at 15 °C. The increase in density during the summer in Brazilian herds may be a result of a greater availability of high-quality forage, since it coincides with the time of largest precipitations (0.45 mm/day in summer vs. 0.01 mm/day in autumn) (28), in which the grass accelerates its growth and increases its nutritional value.

pH is another parameter strongly correlated with SNF. In buffalo milk, pH ranges between 6.57 and 6.84, similar to the pH of conventional cattle milk, but with a slightly higher titratable acidity (17–20°D), reflecting its higher casein and mineral content (study on Murrah buffalo milk) (25). In another study, although no seasonal variations have been observed (since lactic acidity ranged from 0.05–0.20%), Arain et al. (29) mention that the physicochemical characteristics of buffalo milk in Pakistan gradually change according to the postpartum period and the transition from colostrum to normal milk. Colostrum or milk from calved buffaloes at 1 day postpartum had high acidity levels (0.39% or a pH of 6.3), a specific gravity of 1.061, and curd tension of 6.80 cP. In contrast, milk samples at 5 or 6 days postpartum showed decreased values of 0.26%, 1.037, and 1.64 cP, respectively. This is related to higher concentrations of immunoglobulins (18.75%) and minerals (0.20%) in colostrum, which also increase SNF (30).

Curd tension measures milk’s resistance to coagulation or protein denaturation. A study by Liao et al. (21) reported that curd tension of buffalo milk, measured at 25 °C, ranges of 1.5 to 1.8 cP, surpassing that of Kankrej breed conventional cattle milk (1.53–1.65 cP) due to the higher proportion and size of fat globules, which promote intermolecular interactions. This characteristic contributes to a creamier mouthfeel and colloidal stability. From a microstructural and functional perspective, Murrah buffalo milk has larger milk fat globules, with diameters from 4.1 to 4.8 μm, compared to 3.6–4.0 μm in Holstein cow milk (31). Like specific gravity, curd tension is strongly influenced by milk characteristics, such as lipid density. This has been observed in India by Dudi et al. (32), who modified buffalo milk properties to assess protein concentration. The findings showed that a pH between 8.6 and 10.8 or the use of whole buffalo milk increased the concentration of fat globules and protein content, which was associated with a notable increase in curd tension, reaching values of up to 2.02 cP. In contrast, skimmed milk (3% fat), which loses a significant part of its lipid fraction, had a considerably lower viscosity of approximately 1.3 cP. Comparative assessments between riverine (e.g., Mehsana) and swamp-type buffalo milks also indicate higher particle size and viscosity in Mehsana milk than in swamp milk, anticipating different responses to homogenization, fermentation, and storage stability in buffalo dairy products (33). The higher viscosity and larger particle size in the Mehsana breed compared to swamp buffalo could be attributed to genetic selection towards milk production (34). This selection is consistent with larger casein micelles and a higher concentration of colloidal calcium phosphate, which enhances the complexity of the protein particles. Consequently, this structural density increases the internal friction of the milk system, raising its natural viscosity and improving the firmness of the milk gels.

Freezing point (pKa), an important parameter for detecting adulteration and during freezing preservation, differs in buffalo milk when compared to conventional cattle milk. In Murrah buffalo milk, pKa is around −0.552 to −0.528 °C, slightly less negative than that of conventional cattle milk (−0.540 °C), reflecting differences in the concentration of soluble constituents such as sodium and lactose (25). Furthermore, physical treatment of milk, such as boiling and souring, can increase the freezing point, whereas vacuum treatment, cold storage, and the addition of water can decrease this value. This is due to variation in milk composition, high levels of fat, protein, and mineral salts, which decrease the freezing point by restricting crystal formation (21). Mechanistically, process-driven changes in colloidal structure (e.g., homogenization and thermal treatments) redistribute fat globules and modulate micellar dissociation, thereby influencing colligative behavior and the measured freezing point in buffalo milk (21).

A key physicochemical parameter that distinguishes buffalo milk is its buffering capacity, defined as its resistance to pH changes upon the addition of acids or bases. This trait favors pH stability during fermentative and coagulation processes, resulting in advantages in the production of cheeses and yogurts (35). The buffering capacity of Murrah buffalo milk is high (0.042 at a pH of 4.9) due to its high content of casein micelles and colloidal calcium phosphates (CCP). These act as ion reservoirs that neutralize acidity changes. During acidification, CCP solubilizes from the casein micelle, releasing phosphates that consume protons (H+) (36). This process is more prolonged and efficient in buffalo because its micelles are more mineralized (1.12 nM Ca/g and 0.53 nM inorganic phosphate (Pi)/g of casein) and are structurally larger (118 nm) (37), in contrast to cattle milk (0.84 nM Ca/g, 0.36 nM Pi/g, and ∼100 nm, respectively) (36, 38, 39), providing a more robust buffer network. During the acidification process, it is necessary to add an average of 6 mL of nitric acid (HNO_3_) to 1 M of buffalo milk to achieve a pH of 4.0. In contrast, conventional cattle milk achieves this acidity with a lower amount of HNO_3_ (4.5 mL), demonstrating its greater buffering capacity (21). In practice, greater buffering in buffalo milk supports steadier acidification kinetics and firmer gel microstructures in fast-fermented products (e.g., set-type yogurts and fresh cheeses), improving water-holding and curd strength (21).

Another relevant functional and physical quality is heat stability, assessed by the heat coagulation time or the time required to coagulate at 140 °C, where the shorter the time, the lower the thermal stability. Murrah buffalo milk requires approximately 1,563 to 1,581 s, and conventional cattle milk requires 1798 to 1816 s (21, 25). A shorter thermal coagulation time is associated with a faster rate of aggregation upon heating due to the high protein content. Whereas in cattle milk, heat causes a faster denaturation of whey proteins and their subsequent interaction with *κ*-casein (which can destabilize the micelle), in buffalo milk, the lower hydration of the micelles (1.90 g of water/g of dry pellet in buffalo milk vs. 2.24 g of water/g of dry pellet in cow milk) and higher mineralization of casein micelles promote a denser, more compact and hydrophobic structure that resists thermal aggregation (36). For example, in colostrum, the precipitation time increases by more than 50 min during its transition to normal milk, simultaneously with the gradual decrease in protein concentration to 11.85% (29). Similarly, Patel and Mistry (40) reported the association between thermal stability and protein component concentration in Murrah buffalo skim milk, which increases its protein and ash contents by up to 13% during ultrafiltration. Conversely, its thermal coagulation time decreases (4.40 min). Alcohol stability (milk ethanol stability, MES) provides a rapid technological screen that relates to heat-coagulation susceptibility, and it indicates the resistance of casein micelles to dehydration and subsequent aggregation (41). Cross-species comparisons (cow–goat–buffalo) show instability thresholds around ≥ 68% v/v ethanol with species-specific turning points, and buffalo samples often display shorter heat-coagulation times consistent with higher protein, supporting joint consideration of MES and heat stability in ultra-high temperature and high-solids processes (42). The lower degree of hydration, together with the surface charge (free calcium) of buffalo milk, is associated with micelle compaction, which in turn promotes the requirement of a lower alcohol concentration to break the surface water layer (36). Additionally, seasonal and nutritional determinants documented in cows –MES decreasing under feed restriction and improving with cooler seasons and better nutrient intake (43, 44)– are mechanistically plausible in buffalo due to shared micellar and ionic mineral mechanisms. Thus, the mineral balance (Ca/P), modified by seasonal and nutritional factors, dictates the transition of calcium from the colloidal to the ionic state, and this could alter both the MES threshold and heat-coagulation times.

From a technological perspective, the lipid composition of buffalo milk shows a high proportion of saturated fatty acids (~62%), a lower iodine value, and a melting point of fat between 32 and 43 °C, which provides a firm consistency to products such as butter and ghee (17). Likewise, during physical inspections, additional properties include electrical conductivity (around 995 to 1799 μS/cm), moisture content (average of 84.5–90%), and refractive index (in Murrah buffaloes at 40 °C of 1.346 to 1.353) (45). Moreover, organoleptic characteristics, such as the absence of carotenoid pigments, explain its pure white color, which is distinctive in derivatives such as buffalo mozzarella (25), giving them identifiability and specific appeal. In addition, light-induced oxidation is a critical quality constraint: milks and yogurts from swamp-type buffalo exhibit higher photo-oxidative susceptibility than Mehsana, associated with higher free-fatty-acid content and lower intrinsic antioxidant activity; minimizing light and oxygen exposure is therefore advisable in packaging and storage of buffalo dairy products (33).

Physicochemical properties interact closely with the functionality of milk composition, and there could be variations between breeds. For example, SNF and percentages of fat, and protein are significantly higher in crossbreeds of pure water buffalo than in Murrah and Nili-Ravi water buffalo (46). Although the literature is limited, the superiority in SNF concentration can be associated with the heterocyst effect—a phenomenon where the offspring of crosses between pure lines exceed the expected average of their parents in a specific trait (47)—, which is consistent with greater metabolic efficiency in the mammary gland, particularly considering that a moderate heritability (0.26) of milk components (e.g., fat and protein yield and percentage) has been reported in Murrah buffaloes (48). In summary, water buffalo milk combines high nutritional density with physicochemical properties that make it ideal for industrial processing. Its stable pH, high viscosity, density, and buffer capacity are key parameters that determine its technological performance and added value in the dairy industry.

## 3 Nutritional and bioactive composition

### 3.1 Macronutrients

Macronutrients include proteins, fats, and carbohydrates. They represent the majority of milk’s composition and determine its nutritional value, functional properties, processing traits and caseins content (49). Several studies indicate that the percentages of fat, total solids, and especially protein, are higher in buffalo than in conventional cattle milk (50–55). For example, Chakraborty et al. (53) analyzed the composition of raw milk from Murrah buffaloes and compared it with that of Jersey cow milk. The authors found that buffalo milk had higher concentrations of proteins (4.61 ± 0.21% vs. 4.27 ± 0.10%), total solids (15.23 ± 0.25% vs. 14.71 ± 0.33%), fat (6.30 ± 0.12% vs. 5.07 ± 0.10%), and SNF (8.89 ± 0.11% vs. 8.62 ± 0.12), while having lower moisture content (84.18 ± 0.3% vs. 85.89 ± 0.47%).

These findings are consistent with those observed by Yuan et al. (51), who also reported a higher proportion of protein (4.38 ± 0.13% vs. 2.95 ± 0.10%), fat (8.60 ± 0.44% vs. 4.18 ± 0.26%), and total solids (18.88 ± 0.51% vs. 12.70 ± 0.30) in the milk of Italian Mediterranean buffaloes when compared to Chinese Holstein cows (51). These differences could be related to variations in specific metabolic pathways identified through metabolomic analysis, including the metabolism of glycerophospholipid, nicotinate, nicotinamide, glycine, serine, and threonine, and purine, all of which are involved in lipid and protein synthesis and metabolism. However, further research is needed to determine the origin of these differences between these species (51).

### 3.2 Fats

The fat content of processed products defines their functional contribution and directly impacts flavor and rheological properties (56). The lipid fraction of milk is one of the components that most influences its energy density, sensory characteristics, and the processing of dairy products (57).

In this sense, the fat content in the milk of Mediterranean buffaloes is considerably higher than that of cattle (9.86 vs. 3.40%) (25, 58). In buffalo milk, triacylglycerols constitute 98.6% of total lipids, a value slightly higher than that recorded in cattle (97.5%). The diacylglycerol content is also higher in buffalo (0.7 vs. 0.36%) (21). Regarding cholesterol, buffalo milk contains 6.9 mg/100 mL less cholesterol than conventional cattle milk (58, 59).

#### 3.2.1 Fatty acids

**Figure 1:**
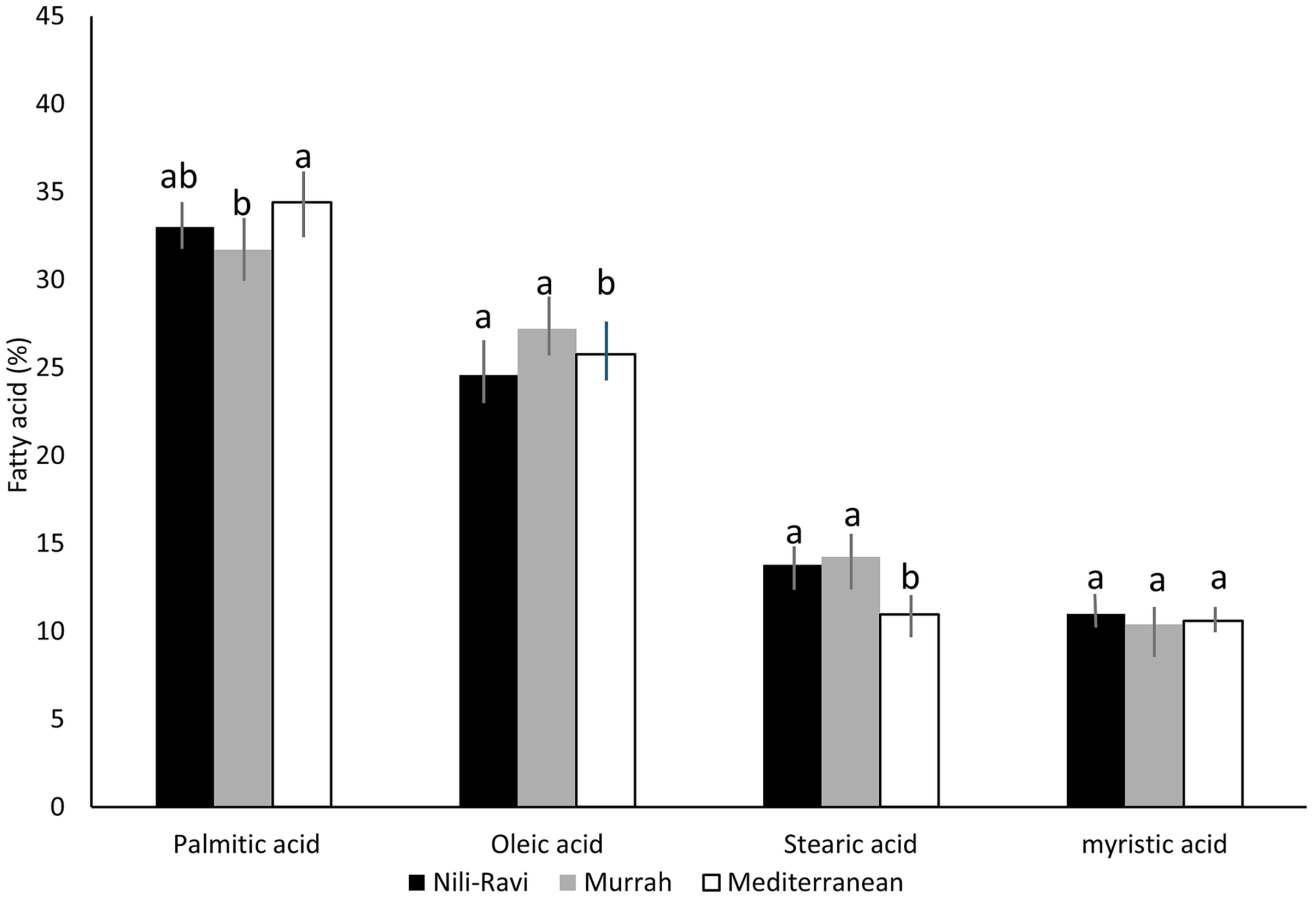
Comparison of fatty acids from Nili-Ravi, Murrah, and Mediterranean buffalo milk. Data are expressed as mean ± standard deviation. Different letters in the same row mean significant differences in fatty acids (*p* < 0.05). Figure created using information from Abdel Hamid et al. (65).

These hydrophobic compounds, derived from aliphatic hydrocarbon chains, influence both the technological properties and the nutritional value of milk and its derivatives (60, 61). Fatty acids influence the shelf life, quality and acceptability of foods and are used to produce desirable textures, melt-away properties, and food structure (62, 63). A study by Varricchio et al. (64) investigated the fatty acid composition in Mediterranean buffalo milk. The results showed that saturated fatty acids represented an average of 65.5% of milk fat, followed by monounsaturated fatty acids (27.0%) and polyunsaturated fatty acids (4.5%). Palmitic acid was the most abundant (30.6%), followed by oleic acid, stearic acid (12.2%), myristic acid (10.6%), and butyric acid (3.4%), representing more than 78% of total fatty acids. The fatty acid profile has been reported with variations in water buffalo breeds, as mentioned by Abdel Hamid et al. (65), whose results are shown in Figure 1. It is worth noting that palmitic acid reached the highest values in Mediterranean buffaloes (34.4 ± 1.5%), followed by Nili-Ravi (33 ± 1.6%) and Murrah (31.7 ± 1.8%), with statistically significant differences. As for oleic acid, the values were 24.6 ± 1.2% for Nili-Ravi, 27.2 ± 1.7% for Murrah, and 25.8 ± 1.7% for Mediterranean, with no relevant differences observed. Stearic acid showed its highest proportion in Murrah (14.2 ± 1%), followed by Nili-Ravi (13.8 ± 0.9%) and Mediterranean (11 ± 0.8%), the latter being significantly lower. Lastly, in myristic acid, no significant differences were detected between breeds, with similar values (11 ± 1.1% in Nili-Ravi, 10.4 ± 1.3% in Murrah, and 10.6 ± 0.8% in Mediterranean). The differences have been associated with the expression of key enzymes during processes such as lipogenesis. In buffalo, polymorphisms have been identified in genes such as diacylglycerol O-acyltransferase 1 (DGAT1) and ESRRG that are related to lipid metabolism and may influence the ratio of saturated to unsaturated fatty acids (66).

#### 3.2.2 Carbohydrates (lactose)

Lactose is the main glucide in milk. It facilitates the action of vitamin D and the intestinal absorption of calcium, magnesium, and phosphorus (67–69). Lactose concentration in buffalo milk is commonly lower than that of cattle. In the Murrah breed, a lactose concentration of 3.69 ± 0.14% has been reported, whereas in the Italian Mediterranean buffalo it is 5.03 ± 0.05%. In comparison, milk from Jersey cows reaches 3.99 ± 0.16% and Chinese Holstein reaches 5.10 ± 0.07% (51, 53). This difference confers unique attributes during the cheese production. With less substrate for lactic acid bacteria, lactic acid production tends to decrease, raising the initial pH of rennet coagulation and syneresis. This could be beneficial for the production of Mozzarella cheese (70–73).

#### 3.2.3 Proteins

The main proteins in milk are casein and whey proteins. Casein accounts for 80% of the proteins in milk and provides stability for processing products such as cheese and yogurt. There are four casein types: αs1-, αs2-, *β*-, and *κ* (74, 75).

Approximately 45% of cattle produce β-casein A1 (linked to gastrointestinal symptoms in some people), while the remaining 55% produce β-casein A2. In contrast, water buffalo produce only β-casein A2, which is better tolerated by sensitive individuals (76, 77). These A1 and A2 variants are distinguished by the presence of an amino acid at position 67, histidine in the A1 variant, and proline in the A2 variant (77).

Caseins are the main protein fraction in buffalo milk and largely determine its coagulation properties. Mediterranean buffalo milk has a high total casein content, averaging values of 49.8 g/L, which favors the formation of compact curds with high solids retention. The proportion and type of casein (especially αs₁-, αs₂-, and *κ*-caseins) directly influence the rennet-coagulation time, the curd-firming time, and curd firmness after rennet addition (9, 78–80).

Likewise, studies have shown that a higher *β*-casein content favors faster coagulation, while αs₁-casein can delay it. Casein αs₂, although less studied, is associated with improved curd firmness, and κ-casein, abundant in this species, guarantees optimal conditions for the action of chymosin and the formation of stable micelles. These particularities explain why buffalo milk has a short coagulation time and high curd firmness, characteristics that contribute to its high yield and quality in cheeses such as mozzarella (78, 79).

### 3.3 Micronutrients

#### 3.3.1 Minerals

Minerals play an essential role in human health, participating in physiological functions such as tissue development, blood coagulation, muscle contraction, and nervous system activity (81). Furthermore, they are crucial in the milk coagulation process, as they intervene in the formation and grouping of casein micelles, which affect rennet coagulation time and curd structure, and consequently influence cheese yield (82–84).

**Figure 2:**
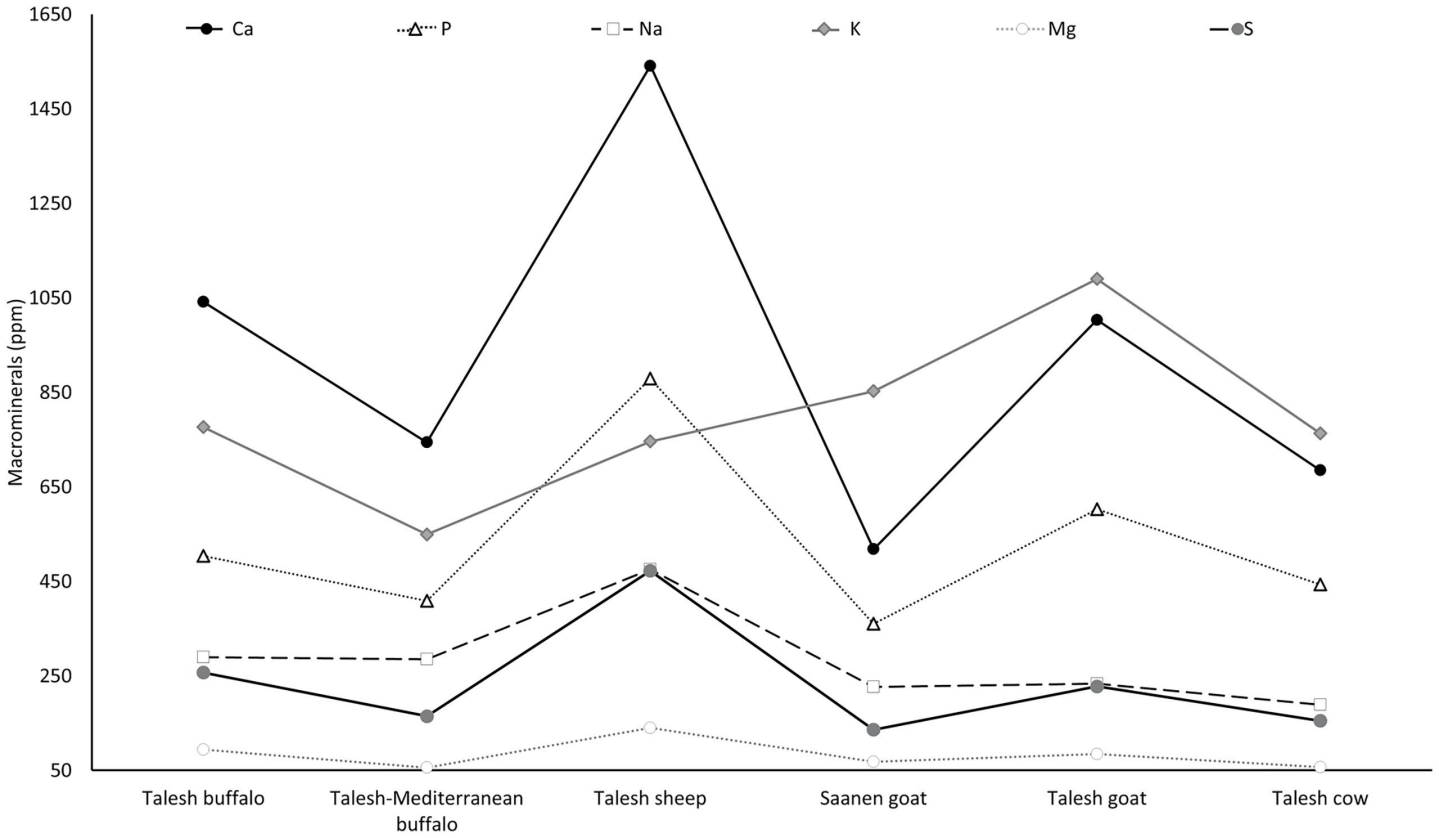
Comparison of macromineral concentrations (ppm) in milk from water buffalo (Talesh and Mediterranean), Talesh sheep, Saanen goat, Talesh goat, and Talesh cow. Data are expressed as means. Figure created using information from Liotta et al. (85).

In this regard, Liotta et al. (85) highlighted the higher calcium (Ca) concentrations (1,042 ppm) of Talesh buffalo milk when compared with Saanen goat (518 ppm), and Talesh goat (1,003 ppm) and Talesh cow (685 ppm) milk (Figure 2). Similarly, higher values of phosphorus (P) (504 vs. 443 ppm), sodium (Na) (290 vs. 189 ppm), potassium (K) (776 vs. 763 ppm), magnesium (Mg) (94 vs. 57 ppm), and sulfur (S) (257 vs. 155 ppm) were recorded in Talesh buffalo milk compared to Talesh cow milk. These differences suggest that Talesh buffalo milk, due to its higher concentration of key minerals, may offer greater nutritional value and technological advantages, such as improved coagulation, higher cheese yield, and distinct sensory characteristics compared to cow and goat milk (85). This is consistent with Lucey and Fox (83), who noted that high Ca and phosphorus levels in milk favor the formation of a firmer curd and increase cheesemaking yield.

These results are similar to those found by Fantuz et al. (86), where it was determined that Mediterranean buffalo milk, compared to Italian Friesian cows, contains higher concentrations of Ca (1,598 vs. 1,153 mg/L), P (1,340 vs. 975 mg/L), Mg (154.6 vs. 104.0 mg/L), zinc (4,978 vs. 3,435 μg/L), iron (321.9 vs. 212.7 μg/L), copper (109.8 vs. 56.0 μg/L). However, in this study, lower concentrations of K (1,021 vs. 1,461 mg/L) and Na (333.9 vs. 391.7) were observed in buffalo compared to cow.

#### 3.3.2 Vitamins

Vitamins E, C, and A play complementary roles in defense against oxidative stress. Vitamin E protects membrane lipids against peroxidation, while vitamin C neutralizes reactive oxygen species and regenerates the active form of vitamin E. Vitamin A modulates the expression of antioxidant genes and helps mitochondrial function (87–89).

A study by Khan et al. (55) showed that the antioxidant profile in pasteurized buffalo milk compared to conventional cattle milk had higher concentrations of vitamin C (0.39 ± 0.04 vs. 0.31 ± 0.06), vitamin A (129.7 ± 1.17 vs. 58.9 ± 1.36), vitamin E (1.21 ± 0.13 vs. 0.17 ± 0.02), selenium (6.25 ± 0.32 vs. 0.92 ± 0.13) and zinc (559.3 ± 2.43 vs. 417.3 ± 3.94). All data are expressed as μg/100 g. This antioxidant profile suggests a higher antioxidant capacity of buffalo milk compared to cattle.

Furthermore, vitamin B12 is essential for deoxyribonucleic acid (DNA) synthesis and cellular energy production (90). Pyridoxine, meanwhile, exhibits a modulatory and selective effect on the production of serotonin and GABA (gamma aminobutyric acid) (91). Buffalo milk has been shown to contain more vitamin B12 (1.28 vs. 1.00 mg/100 g) and pyridoxine (0.44 vs. 0.30 mg/100 g) than conventional cattle milk (50). This superior content of B vitamins positions buffalo milk as a raw material with a more complete nutritional profile.

### 3.4 Bioactive peptides

These proteins exist as micelles and tend to fragment during gastrointestinal digestion, fermentation, and certain non-thermal treatments. In buffalo milk, most of the bioactive peptides come from caseins and, to a lesser extent, from whey proteins (22, 92). During this fragmentation, peptides are released to act locally on the intestinal microbiota, modulating digestive enzymes or influencing immune functions. Some peptides can cross the intestinal mucosa and enter the bloodstream, exerting systemic effects Among the peptides identified in buffalo milk are VLPVPQK, RELEE, EDVPSER, NAVPITPTL, HPHPHLSF, YPFPGPIPN, RNAVPITPTLNR, TKVIPYVRYL, YLGYLEQLLR, and FALPQYLK, which are associated with antioxidant, osteogenic, and antidiabetic properties (21, 22, 92, 93). Beyond classical peptides, buffalo milk nanovesicles (50–200 nm) have been shown to carry microRNAs (e.g., miR-15b, miR-21, miR-27b, miR-125b, miR-155, miR-500) that are more present compared to serum/urine and stable under some household storage conditions (94). These vesicles have bioactive properties, and while their post-ingestion signaling in humans remains to be fully characterized, they present a plausible mechanism for milk-derived epigenetic or cell-regulatory effects.

#### 3.4.1 Angiotensin-converting enzyme (ACE) inhibitor peptides

ACE inhibitor peptides are mainly generated during the proteolysis of caseins in fermentation processes with specific lactic acid bacteria such as *Lactobacillus helveticus*. These peptides have been attributed a blood pressure-regulating effect (95–98). Their effect is mediated by breaking down into specific small, bioactive peptides during digestion or cheese ripening. These peptides bind to ACE’s active sites, preventing the conversion of angiotensin I to angiotensin II, lowering blood pressure (99). In bovine milk, active peptides have an inhibition efficiency ratio for ACE of 0.14%/peptide concentration (100).

In cheeses, αs1-casein contributes fragments such as RPKHPIKHQ and KKYNVPQL, while VRYL has been isolated from αs2-casein. *β*-Casein is represented by peptides such as LRF, APFPEVFGK, YPFPGPIPN, LVYPFPGPINSLPQ, and VRGPFP, which have been detected in varieties such as Gouda, Crescenza, and Manchego (96, 101). In Gouda and Cheddar cheeses, it has been shown that, as ripening progresses, both the concentration of peptides and their hypotensive and antioxidant activity increase (98, 102). Likewise, it was observed that the strain used as a starter culture and the storage conditions determine the final bioactive profile (98).

In fermented milks, *α*s1-casein derivatives predominate, including TTMPLW, LAYFYP, DAYPSGAW, and YP. *β*-Casein yields several bioactive fragments, including IPP, VPP, AVPYPQR, YQEPVL, EMPFPK, and YP again. IPP and YP have been identified from *κ*-casein (96). In whey proteins, α-lactalbumin is derived from the peptide LAHKAL. β-Lactoglobulin produces the peptides GLDIQK and VAGTWY (96).

#### 3.4.2 Caseins

Caseins are organized into macromolecular structures called micelles, whose integrity is maintained by phosphate groups that bind to the α, *β*, and κ fractions (approximately 10, 5, and 1 mol of phosphate per mole of protein, respectively). This variation in phosphate content is crucial for micelle structure and stability, as well as conferring significant antioxidant capacity (103, 104).

#### 3.4.3 Immunomodulatory biopeptides

Among the immunomodulators in buffalo milk is lactoperoxidase, which stands out for its ability to act as an antibiofilm and immunomodulator, protecting against resistant bacteria such as *Salmonella typhi* and *Lysteria monocytogenes* (105). Another immunomodulator present in buffalo milk is *β*-lactoglobulin. This whey protein contains branched-chain and essential amino acids, as well as retinol-binding protein, which could modulate lymphatic processes (50).

Regarding buffalo colostrum, the high concentrations of immunoglobulins, lactoferrin, lactoperoxidase, lysozyme, and proline-rich polypeptides stand out, which favor immune system stimulation and provide passive immunity to the newborn. By fermenting colostrum whey with *Lactobacillus rhamnosus* (C25), bioactive peptides are released, of which 40 with immunomodulatory potential were identified, mainly derivatives of lactotransferrin, β-casein, and albumin (106).

## 4 Biological activity and health benefits

### 4.1 Anti-inflammatory properties

Buffalo milk possesses several functional properties due to its composition, making it suitable for the manufacture of high-quality dairy products (50). Additionally, buffalo milk contains bioactive compounds such as peptides (e.g., *δ*-valerobetaine (δVB) and acetyl-l-carnitine), which participate in immunological, gastrointestinal, and endocrine processes (21, 50, 107). Dairy buffalo milk is rich in L-carnitine and short-chain acylcarnitines, propionylcarnitine (PLC), butyrylcarnitine, isobutyrylcarnitine, and 3-methylbutyrylcarnitine, compounds with anti-inflammatory and neuroprotective effects (108). It has been suggested that the anti-inflammatory effect of acetyl-l-carnitine reduces the production of cytokines (e.g., tumor necrosis factor-*α* [TNF-α], interleukin-1β [IL-1β], and interleukin-6 [IL-6]) and modulates gene expression pathways related to inflammation (21, 109), while PLC is associated with redox balance and plasma-membrane stabilization (110). Likewise, milk from Mediterranean buffaloes has higher linoleic acid concentrations than *Bos taurus* milk, which provides benefits for cardiovascular and energy metabolism (21, 107, 108). Linoleic acid has shown a downregulation of the inducible nitric oxide (NO) synthase, cyclooxygenase-2 (COX-2), and TNF-*α* (111).

Buffalo milk exhibits anti-inflammatory activity by directly targeting pro-inflammatory agents, such as cytokines and reactive oxygen species (ROS) (112). For example, studies have shown that the anti-inflammatory and antioxidant properties of water buffalo milk are due to the presence of the metabolite δVB (107), a betaine present in ruminant milk and meat (113, 114). It has been suggested that δVB inhibits inflammatory signaling pathways by reducing the activation of the nuclear factor kappa (NF-kappa), a regulator of the inflammatory response (115). In this context, D’Onofrio et al. (107) evaluated water-soluble extracts (WSE) from Italian Mediterranean buffalo milk and their effects on inflammation-related cytokine levels, including TNF-α, IL-1β, and IL-6) in endothelial cells. The authors found that cytokine levels significantly reduced to approximately 60, 70, and 80 pg./mL, respectively. Additionally, inhibition of up to 135% on COX-2 and COX 1 (enzymes related to inflammatory processes) was also reported.

Similarly, Rafiq et al. (116) determined the anti-inflammatory activity of WSP of buffalo milk Cheddar cheese on colon cancer cells. After lipopolysaccharide (LPS) stimulation, the authors found that buffalo Cheddar cheese inhibited NO production by 39.5%. NO is a signaling molecule and a pro-inflammatory mediator. Thus, inhibition of NO synthesis through WSP of buffalo milk could be considered a therapeutic option to manage inflammatory diseases (117). In the same type of cells, Wang et al. (118) determined the anti-inflammatory properties of Murrah buffalo and Holstein cow milk on human colon adenocarcinoma cells (Caco-2 cells). Following LPS-induced inflammation stress, the concentrations of TNF-*α*, IL-1β, and IL-6 in the cells co-cultured with milk decreased when compared to the non-treated group (approximately 25, 20, and 20 pg./mL, respectively), suggesting that buffalo milk helps Caco-2 cells to resist LPS-induced inflammation stress.

The anti-inflammatory activity of buffalo milk and whey is closely related to its bioactive composition. In this sense, another anti-inflammatory compound is gangliosides, a molecule that intervenes in metabolic and biochemical functions, improving bone, heart, and gastrointestinal health (22, 119). Colarow et al. (120) mentioned that Italian and Pakistani buffalo milk and colostrum have gangliosides, particularly of the monosialotetrahexosylganglioside (GM1) class, which have not been found in conventional cattle milk (22). Gangliosides can decrease prostaglandin concentrations by up to 80% in colonic epithelial cells, thereby protecting against inflammatory processes (120). Moreover, buffalo milk is rich in lactoferrin (ranging from 0.030 to 0.813 g/kg), a protein with antimicrobial and immune-boosting properties (121). In silico research in other species (camels), lactoferrin has been shown to reduce inflammation by modulating the nuclear factor-kappa B pathway (a group of transcription factors regulating inflammation and cell survival) (122). Thus, although no research has explored this aspect in buffalo milk, it might be considered as an additional anti-inflammatory component.

Another component in buffalo milk is the casein-derived decapeptide YQEPVLGPVR. Sowmya et al. (123) reported the influence of YQEPVLGPVR, present in Indian buffalo milk, on cytokine levels (IL-10, interferon-*γ* [IFN-γ], and transforming growth factor-*β* [TGF-β]) in murine spleen tissues. The addition of the decapeptide decreased the secretion of the pro-inflammatory cytokine IFN-γ (10 vs. 25 pg./mg protein). It increased the concentration of anti-inflammatory cytokines IL-10 (50 vs. 80 pg./mg protein) and TGF-β (40 vs. 50 pg./mg protein) in the cultured splenocytes. Likewise, the role of casein-derived peptides as inflammatory agents in buffalo milk was also addressed in *ex vivo* assays using mice splenocytes, where the hexapeptide YFYPQL decreased the IFN-γ release and increased the levels of IL-10 in splenocyte cultures (124). Moreover, recently, Zheng et al. (112) identified a novel peptide with anti-inflammatory properties in Binglangjiang buffalo fermented milk. The peptide, GG13, successfully suppressed the overproduction of nitrous oxide (NO) and TNF-*α* expression in inflammatory macrophages.

### 4.2 Antioxidant properties

Oxidative stress is associated with the development of various ailments, including cardiovascular diseases, arthritis, inflammation, and cancer (125). Reactive oxygen species (ROS) are part of the normal cellular metabolism and have beneficial physiological effects. However, under pathological conditions, excessive ROS levels induce cell damage and apoptosis, contributing to the progression of degenerative and inflammatory diseases (23).

Buffalo milk has natural antioxidants that might be beneficial in neutralizing free radicals (126–128). Antioxidants are stable molecules that can donate an electron to a free radical, neutralizing it and reducing its potential to cause damage (129). To evaluate the antioxidant potential of milk several methodologies are available. Total Antioxidant Capacity (TAC) measures the ability to neutralize free radicals by donating electrons or hydrogen atoms, and integrates the combined effects of all antioxidants present (55). Reducing Power (RP) reflects the ability to donate electrons and reduce oxidizing agents, interrupting oxidation chain reactions. The DPPH (1,1-diphenyl-2-picrylhydrazyl) free radical scavenging assay uses a stable free radical whose color change allows the quantification of the electron or hydrogen donation efficiency of antioxidant compounds (130). The antioxidant activity in linoleic acid (AALA) evaluates the ability to inhibit lipid peroxidation of this polyunsaturated fatty acid, simulating oxidative processes that occur in biological or food systems (21).

Examples of compounds in buffalo milk that might serve as antioxidants are proteins and monounsaturated fatty acids (55, 131). Vitamin A, E, Zn, and selenium are also antioxidants present in buffalo milk (55). While vitamin E is a peroxyl radical scavenger that protects polyunsaturated fatty acids from damage (132), Zn downregulates ROS production and inhibits the oxidation of DNA, RNA, and proteins (133). Moreover, enzymes such as superoxide dismutase (SOD), glutathione peroxidase, and catalase may also be involved (134). According to D’Onofrio et al. (107), buffalo milk has a high TAC (approximately 4 mM Trolox equivalent). Khan et al. (55) assessed the antioxidant properties of pasteurized and boiled buffalo milk through TAC, RP, DPPH, and AALA, and compared them with those of cow milk values. TAC of raw buffalo milk was higher (58.4%) than conventional cattle (42.1%). RP of buffalo and conventional cattle milk were 13.7 and 6.74, respectively. DPPH activity of boiled milk for buffalo was higher (30.4%) than that of conventional cattle (23.6%). Lastly AALA levels of buffalo and conventional cattle milk were 11.7 and 17.4%, respectively. This indicates a higher antioxidant capacity and AALA levels compared to milk provided by cattle. Additionally, the authors reported that pasteurization and boiling did not affect antioxidant capacity, whereas 3 days of refrigeration decreased it (55).

Wang et al. (118) assessed the antioxidant properties of milk from Murrah buffaloes and Holstein cows in Caco-2 cells. The results showed that, after treatment with H_2_O_2_, almost half of the control cells died; cells co-cultured with milk significantly decreased ROS levels, particularly with buffalo milk compared to Holstein milk (up to 20,538 vs. 15,761). Moreover, SOD and malondialdehyde (MDA) content significantly increased in cells with buffalo milk, approximately to 5 and 8 nmol/10^4^ cells, respectively. The presence of both antioxidant enzymes is considered an oxidative stress marker as they prevent oxidative damage to cells (135). In the same cell type, the cytotoxic effect of WSP from buffalo milk Cheddar cheese was evaluated, resulting in a significant 38.3% decrease in cell viability. Additionally, WSP induced apoptosis (6.3-fold) and increased the early apoptotic cell population (7.3-fold) (116).

Huma et al. (125) also tested the antioxidant potential of WSP of buffalo milk Cheddar cheese. The authors evaluated the impact of WSP extracts on cell viability and ROS production in Caco-2 cells. When comparing buffalo milk with conventional cattle milk, buffalo milk had a greater inhibition of oxidative activity (15.95%) than the second one (15.88%). Moreover, intracellular ROS production in Caco-2 cells decreased significantly with buffalo (17.4%) and conventional cattle milk (11.02%). Similarly, Basilicata et al. (23) tested buffalo-milk dairy products (yogurt, scamorza, grana, mozzarella, ricotta, and ice cream) in an intestinal epithelial cell line (IEC-6), treated with H_2_O_2_, to evaluate their ability to inhibit ROS release. Among the dairy products, buffalo ricotta cheese had abundant *β*-lactoglobulin peptide (YVEELKPTPEGDL). Ricotta cheese also increased antioxidant factors such as SOD and inhibited ROS production (60%). Suppression of ROS production was also associated with higher antioxidant properties of Indian buffalo milk due to its casein-derived decapeptide (YQEPVLGPVR) (ranging from 1 μg to 1 mg/mL) (123).

In another study, the antioxidant potential of Nili-Ravi buffaloes’ colostrum, transition milk, and mature milk was investigated (134). The authors evaluated the Trolox equivalent antioxidant capacity (TEAC) and RP. They found that buffalo colostrum obtained during the first two postpartum milkings had higher TEAC values (107.05 and 91.47 mg/100 g Trolox equivalent, respectively). Similarly, RP was higher at the first lactation (134.03 μg/g Trolox equivalent) and decreased gradually to 60.69 μg/g after the 10th lactation. Thus, the results suggest that buffalo colostrum obtained during the first two days after calving might confer higher benefits to human health due to its enhanced antioxidant potential. Therefore, buffalo colostrum has a higher free radical scavenging activity than conventional cattle milk due to the presence of bioactive peptides and fat globule membranes (134). These results are similar to those reported by Coroian et al. (136) for Romanian buffaloes across the lactation period. The authors reported that the antioxidant capacity of buffalo milk was highest in lactation three (360.1 μg/mL) and four (358.9 μg/mL).

Regarding other dairy products derived from buffalo milk, fermented buffalo milk is called dadih, a type of curd eaten mainly in Indonesia (137). It contains lactic acid bacteria with antioxidant properties, such as *Lactococcus*, *Lactobacillus*, *Bifidobacterium*, *Streptococcus*, and *Pediococcus*. In this regard, Kusumaningtyas and Utami (131) determined the antioxidant activity of dadih using the ABTS (2,2′-Azino-bis(3-ethylbenzothiazoline-6-sulfonic acid), DPPH, and Fe reducing power (ferric reducing antioxidant power) methods. The soluble protein of dadih had a high antioxidant activity against ABTS radical (between 78–90%), and generally, dadih protein had an antioxidant activity of more than 50%. Non-thermal processes already trialed in buffalo matrices (e.g., high-pressure processing and ultrasound) can preserve or modulate antioxidant readouts while ensuring microbiological safety; conversely, ultrasound may increase lipase activity and predispose to lipid oxidation, underscoring the need to validate parameters by product type (21).

The animal’s diet highly influences the antioxidant status of Murrah buffalo milk, as seen using mulberry leaf flavonoids by Li et al. (128). In this study, antioxidant enzymes MDA, total antioxidant capacity, SOD, catalase (CAT), and glutathione peroxidase (GSH-Px) were assessed. Results showed in the treated group compared to control a decrease in serum MDA level (1.39 vs. 5.60 U/mL), as well as lower serum T-AOC (1.85 vs. 3.68 U/mL) and CAT contents (5.29 vs. 7.47 U/mL). These results relate to the role of antioxidant enzymes in the organism’s metabolism, where MDA is a biomarker of oxidative stress and free radical levels. Low MDA levels indicate a healthy antioxidant defense system and reduced oxidative damage (138). Thus, mulberry leaf flavonoids remarkably decrease MDA levels by up to 75%, suggesting beneficial effects on human health. Likewise, Agustinho et al. (139) reported that vitamin E supplementation increased the antioxidant capacity of Murrah × Jaffarabadi buffaloes. Supplementation increased the RP in milk (from 20.67 to 22.50). Moreover, studies have reported that adding 30% alfalfa to the mixed ration of Italian Mediterranean buffaloes increases the antioxidant capacity to 200 nmol, compared with non-supplemented animals (100 nmol), suggesting that diet is an important factor in preserving the bioactive properties of buffalo milk (108).

### 4.3 Energy metabolism coadjutant

Buffalo milk also has anti-diabetic potential due to the presence of protein hydrolysates (casein) and *α*-glucosidase inhibitors, as reported by Hau et al. (24). The authors found that buffalo milk protein hydrolysates had a high degree of hydrolysis (68–100%) and a strong α-glucosidase inhibitory activity (between 55.9–57.1%). These parameters might be related to the management of type 2 diabetes, as in type 2 diabetes, the rate of starch hydrolysis (the breakdown of complex carbohydrates into glucose) is related to postprandial hyperglycemia (high blood sugar after eating) (140). Moreover, fermented buffalo milk has anti-diabetic and ACE-inhibitory properties, as reported by Khakhariya et al. (141), due to the presence of *Limosilactobacillus fermentum* and *Saccharomyces cerevisiae*. These authors found that ACE- inhibitory and lipase- inhibitory activities in buffalo milk were 75.25 ± 1.72 and 61.79 ± 2.14, respectively (141).

In animal models, Youssif et al. (142) compared buffalo fermented milk and one-humped-camel milk in a murine model of diabetes mellitus. The results showed that both fermented milks decreased blood serum glucose levels (from 319.33 ± 0.17 to 224.58 ± 0.84 and from 315.36 ± 0.50 to 117.45 ± 0.67, respectively). Moreover, cholesterol, triacylglycerol, low-density lipoproteins, and very low-density lipoproteins decreased significantly (to 86.38 ± 1.22 mg/dL, 115.13 ± 0.29 mg/dL, 34.11 ± 0.45 mg/dL, and 23.03 ± 0.58 mg/dL) with buffalo milk. The anti-diabetic properties of milk might be related to what Arain et al. (143) mention in a study performed in rabbits and administering camel milk, where the combination of milk with insulin significantly increased organ integrity and stability, and improved glycemic levels. As concerns the levels of liver enzymes (glutamate pyruvate aminotransferase, i.e., GPT, and glutamate oxaloacetate aminotransferase, i.e., GOT), while in diabetic rats both enzymes were elevated, buffalo fermented milk reduced GPT and GOT from (80.95 ± 0.88 to 10.75 ± 0.88 and from 92.0 ± 0.54 to 44.17 ± 0.45 IU/L, respectively). In a type 2 diabetes mellitus model in Wistar rats, Arni et al. (144) tested the effect of dadih (rich in lactic acid bacteria and supplemented with vitamin C), given the role of probiotics in the formation of short-chain fatty acids, which can increase insulin production. The authors found that treated animals had significantly lower glucose concentrations and higher insulin levels.

In the case of an animal model of obesity in mice, the effect of buffalo milk on lipid metabolism was tested by Jiang et al. (52). Animals receiving buffalo milk reduced their levels of triglycerides (1043.79 vs. 988.91 mmoL/L) and glucose (1542.84 vs. 1321.34 mmoL/L). In contrast, Deeba et al. (145) compared the anti-hyperglycemic activity of camel and buffalo milk in rabbits, finding that only camel milk significantly decreased blood glucose levels (as low as 300 mg/dL). However, when evaluating the anti-diabetic properties of buffalo milk in human patients, Wulandari et al. (146) reported significant benefits from consuming buffalo milk curd, a native probiotic source rich in lactic acid bacteria. After receiving buffalo milk curd pudding snacks for one week, glucose levels decreased significantly compared to before treatment (−48.38 ± 40.27 mg/dL). Likewise, cholesterol levels decreased (−41.4 ± 19 mg/dL), as did LDL levels (−27.3 ± 25.09 mg/dL). These results suggest that buffalo milk derivatives may serve as an adjuvant to support proper energy metabolism in humans.

### 4.4 Bone and intestinal health

In general, as noted by Malmir et al. (147), intake of 200 g of milk and dairy products reduces the risk of osteoporosis by 22–37%. When addressing the effects of buffalo milk on bone metabolism, several studies have reported benefits, mainly due to a higher Ca content in buffalo milk compared to other species such as cattle (Sahiwal and Tharparkar) and goats (Alpine x Beetle) (148). This was reported by Amr et al. (149), who compared cattle, camel, and buffalo milk in a Sprague Dawley rat model to evaluate bone health. Buffalo Ca (144 ± 0.71 mg/100 mL) and vitamin D3 (0.40 ± 0.01 mg/100 mL) were higher than in cattle (127 ± 1.41 and 0.35 ± 0.03 mg/100 mL, respectively) but lower than in camel milk (165 ± 2.82 and 3.05 ± 0.02 mg/100 mL, respectively). However, all milks increased serum Ca levels (up to 12.91 mg/dL) and ionized Ca levels (up to 1.54 mmoL/L) in rats.

In a murine animal model of postmenopausal osteoporosis, Reddi et al. (150) evaluated femur anthropometrics and bone mineral density (BMD) to test the anti-osteopenic properties of buffalo milk casein-derived Peptide (NAVPITPTL). It was found that the peptide significantly increased BMD (to approximately 0.3 g/cm^2^) and restored femur microarchitectural parameters. Likewise, NAVPITPTL significantly improved biomechanical bone strength, increased Ca concentrations (10 mg/dL), and suppressed IL-6 (600 pg./mL) and TNF-*α* (500 pg./mL) expression. Therefore, the addition of this peptide to human diets might prevent osteoporosis by restoring the homeostasis of the bone remodeling process and inhibiting bone-resorbing cytokines. It has been reported that NAVPITPTL exhibits anti-osteopenic effect by promoting osteoblast differentiation and activation through the activation of the pAkt signaling pathway, and also prevents oxidative stress associated with bone loss (150). In other studies from the same research group, bioactive peptides such as PEP1 (EDVPSER), PEP2 (NAVPITPTL), PEP3 (VLPVPQK), and PEP4 (HPHPHLSF), derived from buffalo casein, were evaluated in an *in vitro* osteoblast differentiation model (151). Calvarial osteoblast cells cultured in the presence of these peptides for 21 days showed increased the up-regulation of osteoblast differentiation markers such as ALP (alkaline phosphatase**)**, OCN (osteocalcin**)** and COL-1 (type I collagen**)**, and increased mineral deposition.

The bioactive components in buffalo milk are also beneficial for gastrointestinal health due to their influence on intestinal epithelial cells. For example, *Lactobacillus casei* SJRP35, *Leuconostoc citreum* SJRP44, *Lactobacillus delbrueckii* subsp. *bulgaricus* SJRP57 and *Leuconostoc mesenteroides* subsp. *mesenteroides* SJRP58, can produce antimicrobial peptides that also serve as probiotics (152). Buffalo milk contains other lactic acid bacteria such as *Lactiplantibacillus plantarum, Lactococcus lactis, Streptococcus thermophilus, Enterococcus faecium, Lacticaseibacillus casei, Lactobacillus delbrueckii subsp. Bulgaricus, Lactobacillus helveticus, Saccharomyces cerevisiae* with probiotic properties (8). Moreover, *Lactobacillus plantarum* promotes protein synthesis and vitamin and mineral absorption, while *β*-lactoglobulin produces antimicrobial peptides against Gram-positive bacteria, aspects that might help against gastrointestinal diseases (50).

Additionally, buffalo milk has potential applications for people with lactose intolerance due to its low levels of lactose and to the presence of several lactobacilli that serve as probiotics (9, 153). For example, Sheehan and Phipatanakul (153) reported a clinical case of an 11-month-old human patient allergic to conventional cattle milk extract with immunoglobulin E (IgE) levels up to 4.99 kU/L. After the introduction of water buffalo milk-based yogurt (skin testing negative), the allergen-specific IgE levels to conventional cattle milk reduced to 1.34 kU/L. More recently, dairy products prepared with lactose-hydrolyzed buffalo milk (Khoa) have been reported to have low lactose levels (fresh buffalo milk: 4.95 ± 0.10% vs. hydrolyzed milk: 0.23 ± 0.02%), which might benefit human health in terms of suitability for people with allergies/intolerances, although further studies in humans are required (154).

### 4.5 Anticancer applications

The antineoplastic properties of Italian Mediterranean buffalo milk have been reported by D’Onofrio et al. (155). The authors used milk extracts with δVB to perform a cell viability assay of human colorectal cancerous cells (adenocarcinomas), finding that 40% of milk extract induced cytotoxicity and reduced cell viability after 72 h of incubation (from 1 optical density 570 nm to 0.6 optical density 570 nm). Cell survival decreased to 50.2% with 2 mM of δVB and 40% milk. The same research group found in *in vitro* studies that δVB has anticancer activity against human oral squamous cell carcinomas (156). In this study, milk extracts of Italian Mediterranean buffaloes reduced cancer cell proliferation by 36.4%. However, when comparing buffalo with goat and sheep milk, Niero et al. (126) reported that total antioxidant activity in sheep milk was higher (7.78 mmol/L of Trolox equivalent).

El-Bahgy et al. (157) investigated buffalo milk and its effect on the growth and viability of HeLa cells, a cervical cancer cell line. The study found that buffalo milk reduced the apoptosis of HeLa cells and increased their viability (3.59 ± 0.0195). Moreover, Caspase-3 and Caspase-9 were stimulated, which suggests that buffalo milk increases the proliferation and viability of HeLa cells by decreasing apoptosis and cell viability. On another type of cancer cells (CaCo2 cells), De Simone et al. (158) used protein-derived peptides released in buffalo Mozzarella cheese to research its cytomodulatory effect. Treatment of CaCo2 cells with purified peptide from buffalo cheese reduced mitochondrial superoxide anion (from 6.2 to 2.5%) and resulted in a 5-fold decrease in cyclin A expression. A reduction in cyclin A levels is often related to cellular growth inhibition, which might be beneficial for this type of cancer (159).

Rizzo et al. (160) found that Mediterranean buffalo milk whey can cause necroptosis and apoptosis in a mouse model of colorectal cancer. Both forms of programmed cell death were accompanied by expression of caspase-3, a cysteine protease associated with pro-survival cellular roles (161). In a similar colorectal carcinoma mouse model, Cacciola et al. (162) reported the effect of delactosed Mediterranean Italian dairy buffalo milk whey (DMW). Pre-treatment with DMW reduced the percentage of mice showing aberrant crypt foci (early precancerous lesion characteristics of colon carcinoma) (treated 33.3 vs. control 75%). Moreover, increases in blood levels of butyric acid (treated 0.2 vs. cancer model 0.4 μg/mL) and cancer diagnostic markers (5-methylcytidine and glycerophosphocholine) were recorded. Furthermore, buffalo milk exerted cytotoxic effects on two human colorectal carcinoma cell lines (HCT116 and HT29), reducing the cell viability by up to 79.5 and 98.9%, respectively, at 20% concentrations.

Buffalo milk fat globule membranes (MFGM) also induced apoptosis and reduced the viability of HT-29 cells in Ji et al.’s (20) study. Results showed that the MFGM content of buffalo milk was 0.15% with 416.5 mg/g protein content, and that 100 μg/mL of MFGM reduced cell viability by 71.26%. Similarly, Ramírez et al. (163) reported the anticancerogenic effect of Murrah × Mediterranean milk on rats receiving carcinogen treatment. The rats treated with functional milk had fewer tumors (20%) and fewer dysplastic crypt foci (70.83%) than rats receiving only water. These percentages significantly increased after 240 days of treatment to 77.78 and 88.89%, respectively. The authors concluded that the conjugated linoleic acid (CLA) and omega-3 fatty acids present in buffalo milk might be related to anticancer properties. Thus, the presence of several bioactive compounds in buffalo milk makes them a potential source for use in functional food product development as anti-inflammatory, antioxidant, and anti-cancer agents.

## 5 Limitations and challenges of the health benefits of water buffalo milk

It has been observed that water buffalo milk has potential health benefits due to its participation in inflammation, oxidative stress, bone health, and even in tumoral processes. However, most of the research regarding buffalo milk as a functional food has been performed *in vitro* or in animal models. This represents a limitation where clinical trials in humans are required to fully comprehend the benefits that water buffalo can provide for different human ailments.

Other aspects that need to be addressed in further studies are the effect of animal-inherent attributes or their management. For example, the content of certain milk compounds can change according to the parity (e.g., lactoferrin content has low values in the first parity) (164). This influences milk composition and, subsequently, directly affects the beneficial composition of buffalo milk. Other characteristics, such as the impact of breed, diet, and lactation stage or long-term health outcomes in human consumers, remain to be studied.

**Figure 3:**
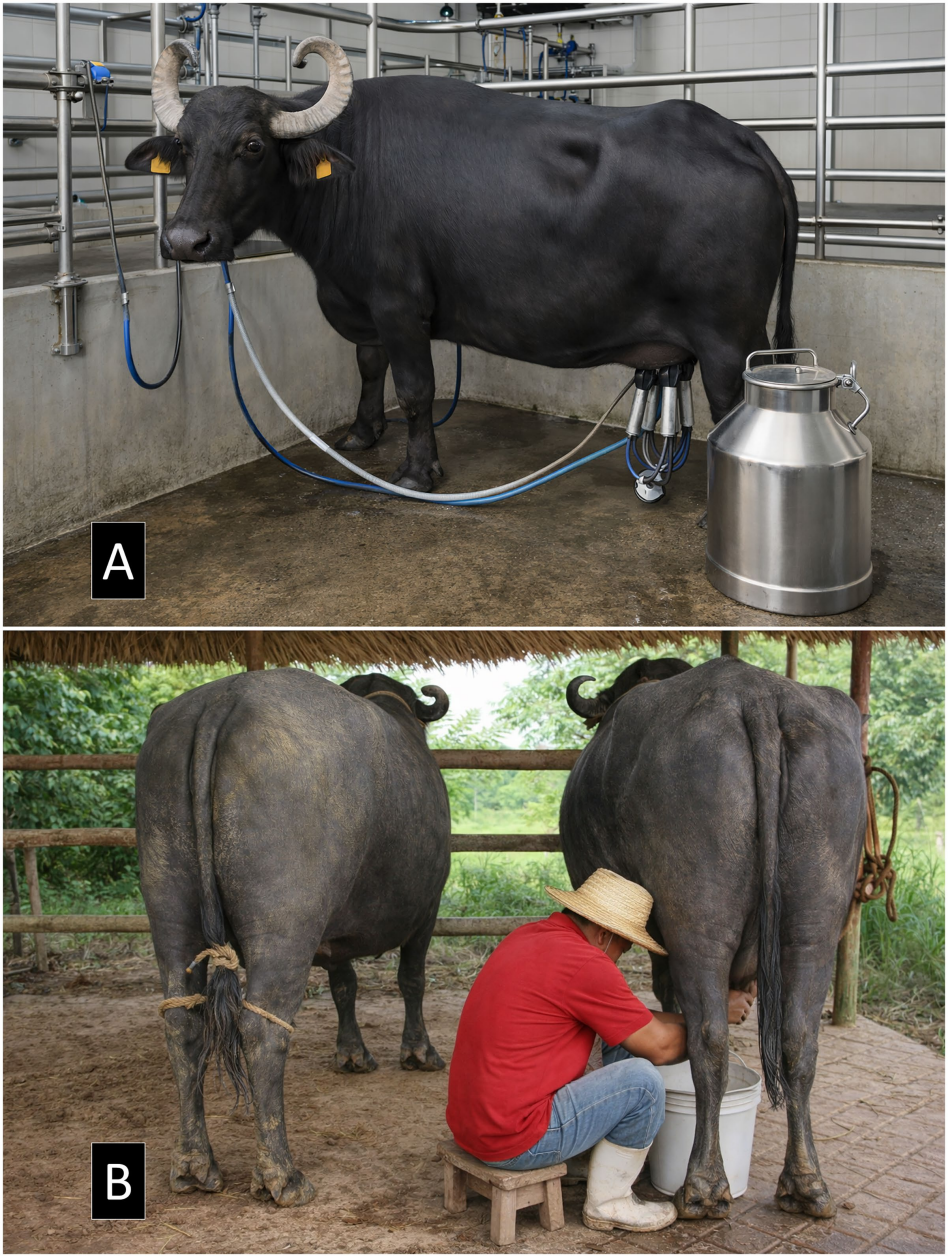
Comparison of milking systems in dairy water buffaloes (*Bubalus bubalis*). **(A)** Mechanized milking system under controlled conditions, characteristic of intensive and technified production systems; **(B)** Manual milking, commonly used in grazing-based or dual-purpose systems. These systems differ not only in milk extraction technique, but also in management practices, hygiene conditions, feeding strategies, and animal welfare. In particular, grazing-based systems, often associated with manual milking, may involve the use of improved pastures that modulate the rumen microbiome and, consequently, influence milk composition and quality. Therefore, differences in milk quality should not be attributed solely to the type of milking, but rather to the combined effects of the production system and associated management practices.

Future research should also consider evaluating the benefits of buffalo milk against other non-bovine species, such as donkeys and camels, which have also shown potential to treat oxidative-stress-related disorders, inflammatory events, and metabolic imbalances (165). Finally, an important aspect when considering water buffalo milk or its derivatives to aid human health is the effect that technological procedures might have on its anti-inflammatory or antioxidant activity. While this has been studied in other species, such as camels (166, 167), future research could explore and achieve processing optimization to prevent the alteration of the chemical composition of buffalo milk and increase its functionality. For example, studying whether non-thermal processing or cheese yield optimization techniques might influence the properties of water buffalo milk related to health benefits. An additional limitation of this review is the lack of explicit consideration of production systems and management conditions as determinants of milk quality. Although the literature included provides a comprehensive description of the physicochemical and nutritional characteristics of buffalo milk, it does not directly address how different milking systems may influence these attributes. It is important to note that the milking method is often closely linked to the production system: pasture or dual-purpose systems are more frequently associated with manual milking, whereas intensive, technified, and confined systems typically employ mechanized or automated milking (13) (Figure 3). These differences involve not only variations in milking technique, but also in hygiene practices, feeding strategies, animal stress levels, and overall management routines, all of which can significantly influence milk composition, microbiological quality, and functional properties. In this context, the feeding system also plays a key role, as the use of improved pastures can modulate the rumen microbiome by altering the availability of fermentable substrates, thereby influencing the production of metabolites such as volatile fatty acids and the synthesis of compounds that may be transferred to milk. Therefore, potential differences attributed to the type of milking should be interpreted with caution, as they may actually reflect the combined effects of the production system, diet, and management practices. Future research is needed to disentangle and independently evaluate these factors in order to better understand their impact on buffalo milk quality (13) (Figure 3).

## 6 Conclusion

Water buffalo (*Bubalus bubalis*) milk has a high nutritional density and physicochemical properties that make it an optimal raw material for cheese-making and processing in the food industry. Its constant pH, remarkable viscosity, high density, and excellent buffering capacity, combined with a distinctive freezing point, represent essential factors that define its technological performance and provide additional value within the dairy sector. In this context, the present analysis shows that buffalo milk not only stands out for its technological properties but also displays a nutritional and bioactive profile superior to that of conventional cattle (*Bos taurus* and *Bos indicus*) milk, characterized by higher concentrations of fat, protein, total solids, essential vitamins and minerals, as well as peptides with antioxidant, antihypertensive, and immunomodulatory properties. These traits have been shown to have health benefits such as anti-inflammatory, antioxidant, anti-diabetic, and antineoplastic activity. However, most research addressing health benefits related to buffalo milk has been performed in animal models, which limits its clinical use on actual human patients. Thus, further research must consider evaluating the effect of buffalo milk in human individuals to comprehensively understand the public health implications of buffalo milk.
